# From microbiome collapse to recovery: a roadmap for microbiome-informed grassland restoration under global change

**DOI:** 10.3389/fmicb.2026.1741287

**Published:** 2026-01-27

**Authors:** Xu Qiao, Xu Yan, Cui Dong, Lin Tao, Aishajiang Aili, Abdul Waheed

**Affiliations:** 1 College of Resources and Environment, Yili Normal University, Yining, Xinjiang, China; 2 Institute of Resources and Ecology, Yili Normal University, Yining, Xinjiang, China; 3 Ecological Water Conservancy Research Center of Xinjiang Uygur Autonomous Region, Ürümqi, China; 4 Xinjiang Comprehensive Land Consolidation and Rehabilitation Center, Ürümqi, China; 5 Technology Innovation Center for Ecological Monitoring and Restoration of Desert-Oasis, MNR, Ürümqi, China; 6 State Key Laboratory of Desert and Oasis Ecology, Xinjiang Institute of Ecology and Geography, Chinese Academy of Sciences, Ürümqi, China

**Keywords:** ecosystem resilience, grassland ecosystems, grassland restoration, microbial diversity, microbiome engineering, soil–plant interactions

## Abstract

Grassland ecosystems depend on soil- and plant-associated microbiomes that regulate nutrient cycling, soil structure formation, plant health, and stress tolerance. This review synthesizes recent progress on how grassland microbiomes are assembled across rhizosphere, endosphere, and bulk soil niches, and how degradation drivers (e.g., overgrazing, drought, salinization, and nutrient enrichment) disrupt microbial diversity, network stability, and functional guilds, often shifting communities toward reduced mutualist capacity and greater disease risk. We then evaluate restoration strategies that aim to re-establish beneficial microbial functions through practices such as organic amendments, inoculation with mycorrhizae or plant growth–promoting microbes, and management approaches that promote habitat recovery and microbial recolonization. Despite rapid advances in sequencing and observational studies, major gaps remain: (i) limited causal evidence linking microbiome changes to process rates (e.g., nitrification, phosphorus mobilization) across field gradients; (ii) underrepresentation of soil viral ecology and its consequences for microbial regulation and ecosystem function; (iii) inconsistent persistence and context-dependence of introduced inoculants; and (iv) a lack of standardized, outcome-oriented indicators for “restoration-ready” microbiomes. Future research should integrate multi-omics with process-based measurements and long-term field experiments, develop locally adapted microbial consortia with monitoring of non-target effects, and strengthen risk assessment and governance frameworks to enable safe, scalable microbiome-informed grassland restoration under global change.

## 1 Introduction

Grasslands constitute one of the largest and most ecologically significant terrestrial biomes, encompassing approximately 40% of the Earth’s land surface (Dengler et al., 2020). These ecosystems perform a wide array of ecosystem services, including but not limited to carbon sequestration, biodiversity maintenance, soil stabilization, water regulation, and the provisioning of forage for livestock functions that are critical for both natural systems and socio-economic stability (Michalk et al., 2019). Despite their ecological and economic importance, grasslands are undergoing accelerated degradation on a global scale (Avishek and Kumar, 2021). Anthropogenic pressures such as overgrazing, agricultural intensification, land-use change, climate variability, and biological invasions are among the principal drivers contributing to the decline of grassland health and functionality (Lyons et al., 2023).

In recent years, there has been a paradigm shift in the understanding of the biological mechanisms underlying grassland stability, with increasing attention focused on the role of microbiomes, complex assemblages of bacteria, fungi, archaea, and viruses inhabiting the soil and plant tissues (Sokol et al., 2022). Soil microbial communities are central to terrestrial biogeochemical cycling, driving critical processes such as organic matter decomposition, nitrogen fixation, phosphorus solubilization, and the formation of soil aggregates that underpin structural integrity (Jagadesh et al., 2024). Simultaneously, plant-associated microbiomes, particularly those in the rhizosphere and endosphere, exert significant influence on plant health by modulating nutrient uptake efficiency, hormonal signaling, pathogen resistance, and abiotic stress tolerance (Govindasamy et al., 2018). For example, Vesicular-arbuscular mycorrhizal fungi (VAMF) form mutualistic symbioses with the majority of grassland plant species, enhancing nutrient acquisition (notably phosphorus) and water retention capacity, especially under drought conditions (Abdalla et al., 2023). Similarly, diazotrophic bacteria such as *Rhizobium* and *Bradyrhizobium* contribute significantly to the nitrogen economy of grasslands, which are often nitrogen-limited systems (Werner et al., 2015; Jia et al., 2021).

The functional contributions of microbiomes extend beyond soil fertility and plant productivity; they are increasingly recognized as key determinants of ecosystem resilience in the face of environmental perturbations (Shah et al., 2022). Microbial community structure and function are highly sensitive to environmental change, and shifts in microbial assemblages can either enhance ecological buffering capacity or accelerate degradation trajectories (Philippot et al., 2021). Degraded grasslands are frequently characterized by reduced microbial biomass and diversity, breakdown of mutualistic interactions, and dominance of opportunistic or pathogenic taxa, leading to diminished soil quality, lower primary productivity, and impaired recovery potential (Zhang et al., 2024). Conversely, restoration initiatives that promote the reinstatement of beneficial microbial consortia either through passive recovery, active microbial inoculation, or the reestablishment of native vegetation have shown potential to restore critical soil functions and facilitate plant community reassembly (Graham and Knelman, 2023; Xu et al., 2025).

However, despite the proliferation of studies examining the ecological roles of microbiomes, several fundamental knowledge gaps remain. The context-dependency of microbial responses across spatial gradients, seasonal dynamics, soil types, and disturbance regimes is poorly understood. Furthermore, the mechanisms through which microbiomes influence or constrain restoration trajectories particularly under compounding anthropogenic pressures—have yet to be systematically synthesized (Chomel et al., 2022). Emerging interest in microbiome engineering, such as the use of targeted microbial inoculants or amendments to steer community composition and function, presents new opportunities for grassland restoration, but these approaches require careful evaluation of efficacy, scalability, and ecological risk (Peddle et al., 2025). Given the urgency of reversing grassland degradation and promoting sustainable land management, a comprehensive synthesis of current knowledge on microbiome-mediated processes is both timely and necessary.

In this review, we systematically assess the current state of knowledge regarding the role of microbiomes in grassland ecosystem functioning and restoration. We first synthesize recent findings on microbial community composition, diversity, and functional traits relevant to grassland soils. We then evaluate how disturbances disrupt soil–plant–microbe interactions and examine the cascading consequences for ecosystem stability. Next, we review microbial-based restoration strategies, including emerging technologies and field-tested approaches, highlighting both their promise and limitations. Finally, we identify priority research directions to advance the integration of microbiome science into grassland management frameworks. By elucidating the microbial underpinnings of grassland degradation and resilience, this synthesis aims to support the development of evidence-based, microbiome-informed restoration practices.

## 2 Microbial diversity and function in grasslands

Soil microbial communities in grassland ecosystems are integral to ecosystem function, underpinning core processes such as nutrient cycling, organic matter turnover, soil structure formation, and plant community regulation (Yang et al., 2025). These communities comprise diverse and dynamic assemblages of bacteria, fungi, archaea, and viruses, occupying distinct ecological niches within the soil matrix, rhizosphere, and plant tissues (Reynolds et al., 2003; Lu, 2024). Their taxonomic composition, functional capacities, and spatial distribution reflect the influence of biotic and abiotic filters, including plant species composition, edaphic characteristics, and climatic conditions (Teague and Dowhower, 2022).

**Figure 1:**
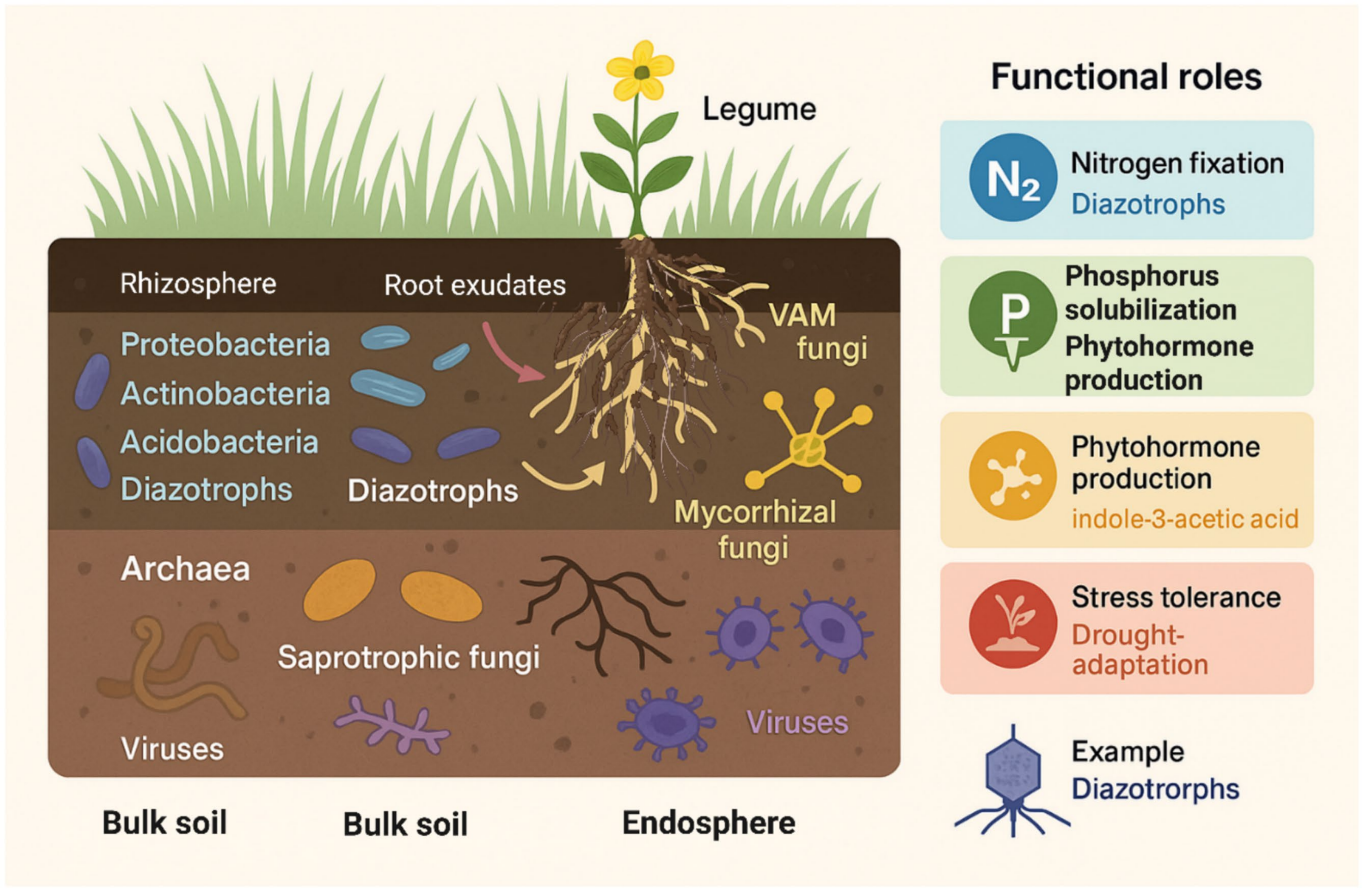
Microbial diversity and functional roles in grassland ecosystems. Illustration of major microbial groups and their ecological functions across the rhizosphere, bulk soil, and endosphere in grasslands. The rhizosphere hosts abundant bacterial taxa (*Proteobacteria*, *Actinobacteria*, and *Acidobacteria*) and diazotrophs that are stimulated by root exudates, along with VAMF that enhance nutrient uptake. Bulk soil contains archaea involved in nitrification, saprotrophic fungi in organic matter decomposition, and diverse viruses influencing microbial community dynamics. The endosphere harbors mycorrhizal fungi and beneficial endophytes that contribute to plant growth and stress tolerance. Functional roles include nitrogen fixation, phosphorus solubilization, the production of phytohormones (such as indole-3-acetic acid), and drought adaptation.

Microbial diversity in grassland soils is particularly pronounced in the rhizosphere, the narrow zone of soil influenced by root exudates, where microbial abundance and metabolic activity surpass those of bulk soils (Gregory, 2006). Root exudates, consisting of low-molecular-weight carbon compounds, amino acids, and signaling molecules, selectively enrich copiotrophic bacterial taxa such as *Proteobacteria*, *Actinobacteria*, *Acidobacteria*, and diazotrophs, and promote symbiotic associations with vesicular–arbuscular mycorrhizal fungi that enhance nutrient acquisition and stress tolerance (Figure 1). This rhizosphere-centric microbial enrichment is not only central to plant performance but also contributes to plant–soil feedbacks that shape vegetation dynamics and successional trajectories (Table 1).

**Table 1:** Key microbial taxa in grasslands, their functions, and roles in ecosystem function, degradation, and recovery.

Microbial group	Representative taxa	Primary functions	Positive contributions to grassland function	Negative or degradation-linked effects	References
Nitrogen-fixing bacteria	*Rhizobium*, *Bradyrhizobium*, *Azotobacter*, *Frankia*	Biological nitrogen fixation	Enhance N availability; support legume growth; promote plant productivity	Decline under overgrazing or soil compaction; leads to nitrogen limitation and reduced vegetation cover	Alomari et al. (2024)
Phosphorus-solubilizing bacteria	*Pseudomonas*, *Bacillus*, *Penicillium*	Mobilize insoluble P via organic acid production	Increase P bioavailability in poor soils; improve plant nutrition	Absence or suppression under land-use change or fertilization disrupts P cycling	Rawat et al. (2021)
Arbuscular mycorrhizal fungi (AMF)	*Glomus*, *Rhizophagus*, *Funneliformis*	P uptake; soil aggregation; water acquisition	Improve drought tolerance, nutrient acquisition, and soil structure; foster plant diversity	Severely reduced by intensive land-use or invasive plants; impairs root function and ecosystem resilience	Wahab et al. (2023)
Endophytic fungi and bacteria	*Neotyphodium*, *Epichloë*, *Azospirillum*	Modulate stress responses; hormonal signaling; pathogen defense	Improve plant tolerance to drought and salinity; enhance root development	Loss of mutualists increases susceptibility to stress and pathogens	Chaffai et al. (2024)
Ammonia-oxidizing archaea (AOA)	*Nitrososphaera*, *Nitrosopumilus*	Nitrification under low N conditions	Regulate N availability in semi-arid and nutrient-limited soils	Sensitive to pH or disturbance; disruption causes N imbalance and losses	Wang et al. (2021)
Decomposer fungi	*Aspergillus*, *Mucor*, *Trichoderma*	Breakdown of organic matter; carbon cycling	Contribute to soil organic matter turnover and fertility	Disrupted under drought or compaction; reduces SOM formation and microbial activity	Satya et al. (2025)
Pathogenic fungi and bacteria	*Fusarium*, *Rhizoctonia*, *Pythium*	Cause root diseases and biomass loss	Natural antagonists can regulate them; their absence reflects microbial balance	Overrepresented in degraded systems; reduce plant vigor and ecosystem recovery potential	Jambhulkar et al. (2015)
Plant growth-promoting rhizobacteria (PGPR)	*Bacillus*, *Pseudomonas*, *Streptomyces*	Produce phytohormones; siderophores; stress alleviation	Stimulate plant growth, nutrient uptake, and abiotic stress resistance	Their decline results in poor plant performance and increased susceptibility to environmental stressors	Azeem et al. (2023)
Viruses (e.g., bacteriophages)	Diverse bacteriophages	Regulate microbial populations via lysis; gene transfer	Control microbial community structure; potential role in resilience and adaptation	Role underexplored; viral lysis may reduce keystone microbial populations in stressed systems	Huang et al. (2024)

Table is compiled and synthesized from published studies/reviews on grassland microbiomes and restoration.

Beyond the soil-root interface, the endosphere serves as an important microbial habitat. Endophytic microorganisms those inhabiting plant internal tissues without causing harm play multifaceted roles in modulating host physiology. For instance, endophytic fungi such as *Neotyphodium* spp. are prevalent in temperate grasslands and have demonstrated capacities to modulate drought responses in host grasses by regulating stomatal conductance and osmotic adjustments (Munir et al., 2022). Similarly, bacterial endophytes, including *Azospirillum brasilense*, enhance root architecture, promote hormonal signaling, and facilitate nitrogen uptake, particularly under nutrient-limiting conditions (Lee et al., 2021). These endophytic communities encompass diverse fungi and bacteria with complementary functional attributes that contribute to plant growth and stress tolerance (Figure 1).

From a functional perspective, distinct microbial taxa contribute disproportionately to key biogeochemical transformations. Bacterial phyla such as *Proteobacteria*, *Actinobacteria*, and *Acidobacteria* dominate many grassland soils, with high functional diversity in carbon degradation pathways, nitrogen cycling, and phosphorus solubilization (Bashan and de-Bashan, 2010). Diazotrophic bacteria, particularly *Rhizobium*, *Bradyrhizobium*, and free-living nitrogen fixers like *Azotobacter*, as well as vesicular–arbuscular mycorrhizal fungi, collectively provide nitrogen inputs, enhance phosphorus mobilization, regulate phytohormone production such as indole-3-acetic acid, and improve drought resilience in host plants (Figure 1). Mycorrhizal fungi, predominantly VAMF, are also widespread in grasslands and enhance phosphorus acquisition, modulate hormone signaling, and improve plant drought tolerance making them essential mutualists in semi-arid and temperate ecosystems (Yang et al., 2022). In addition to these primary decomposers and symbionts, microbial guilds involved in sulfur cycling, methane oxidation, and secondary metabolite production also contribute to the ecological multifunctionality of grasslands. Viral assemblages influence microbial population dynamics through predation and lysis and mediate horizontal gene transfer (Muindi, 2021). Fungal communities, including saprotrophs and dark septate endophytes, perform essential roles in organic matter decomposition and stress mitigation under environmentally challenging conditions (Figure 1).

Despite mounting evidence of the ecological importance of microbiomes in grasslands, substantial heterogeneity exists across biogeographical regions and grassland types. Arid, temperate, and alpine grasslands harbor distinct microbial assemblages with variable functional potentials, shaped by differences in climate, soil pH, vegetation structure, and land-use history (Liang et al., 2025). Furthermore, land management practices, such as grazing intensity, fire regimes, and fertilization, impose strong selective pressures that alter microbial community composition and function (Arunrat et al., 2024). These variations underscore the need for regionally specific microbial assessments to inform context-appropriate restoration interventions (Harman et al., 2021).

Fungi, particularly VAMF fungi, play a pivotal role in nutrient dynamics within grassland ecosystems. VAMF form extensive hyphal networks that enhance the uptake of phosphorus and micronutrients while simultaneously improving soil aggregation through the production of glomalin, a glycoprotein essential for maintaining soil structure (Wahab et al., 2023). In degraded grasslands, the re-establishment of VAMF networks has been linked to enhanced plant colonization and increased soil stability, highlighting their critical role in ecosystem recovery (Wang, 2017). These contributions are supported by their prominent representation among root-associated functional groups in grassland microbiomes (Figure 1).

Archaea, although typically less abundant than bacteria, can exert disproportionate control over key biogeochemical processes, particularly nitrification. In semi-arid grasslands, ammonia-oxidizing archaea (AOA) such as *Nitrososphaera viennensis* may dominate ammonia oxidation under low ammonium availability, where archaeal ammonia oxidizers can outcompete bacterial counterparts (Farooq et al., 2022). By regulating the conversion of NH₄^+^ to NO₃^−^, AOA directly influence plant-available nitrogen and downstream pathways of nitrogen loss (e.g., leaching and gaseous emissions). These archaeal taxa occur across bulk soil and rhizosphere niches, highlighting the need to consider archaeal indicators when evaluating nitrogen cycling dynamics in restoration contexts (Figure 1).

Viruses, particularly bacteriophages, are increasingly recognized as regulators of microbial community structure and function. By lysing specific bacterial populations and facilitating horizontal gene transfer, viruses indirectly affect nutrient cycling and microbial diversity (Chevallereau et al., 2022). Recent grassland studies indicate that soil viruses can influence microbial community composition and function via host-specific lysis, lysogeny–lysis switching under environmental pulses, and gene exchange that alters microbial traits and metabolic potential (Graham et al., 2024). Viral life-history strategies (e.g., predominantly lytic versus temperate/lysogenic dynamics) may differentially regulate bacteria, fungi, and archaea, thereby shaping decomposition, nutrient turnover, and microbial network stability during degradation–restoration transitions, thereby shaping decomposition, nutrient turnover, and microbial network stability during degradation–restoration transitions (Liang et al., 2024). In grassland soils, viral dynamics remain underexplored but may represent a hidden force shaping microbial-mediated ecosystem functions (Figure 1).

The functional traits of these microbial communities are closely linked to grassland health. Traits such as nitrogen fixation, phosphorus solubilization, phytohormone production (e.g., indole-3-acetic acid), and the synthesis of antimicrobial compounds directly enhance plant growth, nutrient uptake, and resistance to diseases (Liang et al., 2025). For example, phosphorus-solubilizing bacteria isolated from grassland rhizospheres have been shown to enhance plant biomass production by mobilizing insoluble phosphate sources (Widdig et al., 2019). Similarly, the production of exopolysaccharides by drought-adapted soil bacteria improves soil water retention, benefiting both microbial and plant communities during dry periods (Gebauer et al., 2022). These processes are represented within multiple functional guilds in grassland ecosystems (Figure 1).

Beyond nitrogen-cycling guilds such as AOA, other microbial functional groups (e.g., mycorrhizal fungi) contribute to stress buffering and restoration outcomes. Moreover, the ability of mycorrhizal fungi to ameliorate plant stress under saline and nutrient-poor conditions underscores the multifunctionality embedded within grassland microbiomes (Ndoye et al., 2024). Rather than reflecting taxonomic richness alone, microbial diversity represents complementary functional traits that support plant establishment and soil recovery following disturbance. Accordingly, restoration strategies that incorporate microbiome management, including targeted microbial inoculation and organic amendments, may accelerate recovery by enhancing nutrient acquisition, improving soil structure, and strengthening plant stress tolerance under changing environmental conditions (Nizamani et al., 2024).

## 3 Soil–plant-microbe interactions in grasslands

### 3.1 Mechanisms of communication and symbiosis (e.g., rhizosphere interactions, mycorrhizal associations)

**Figure 2:**
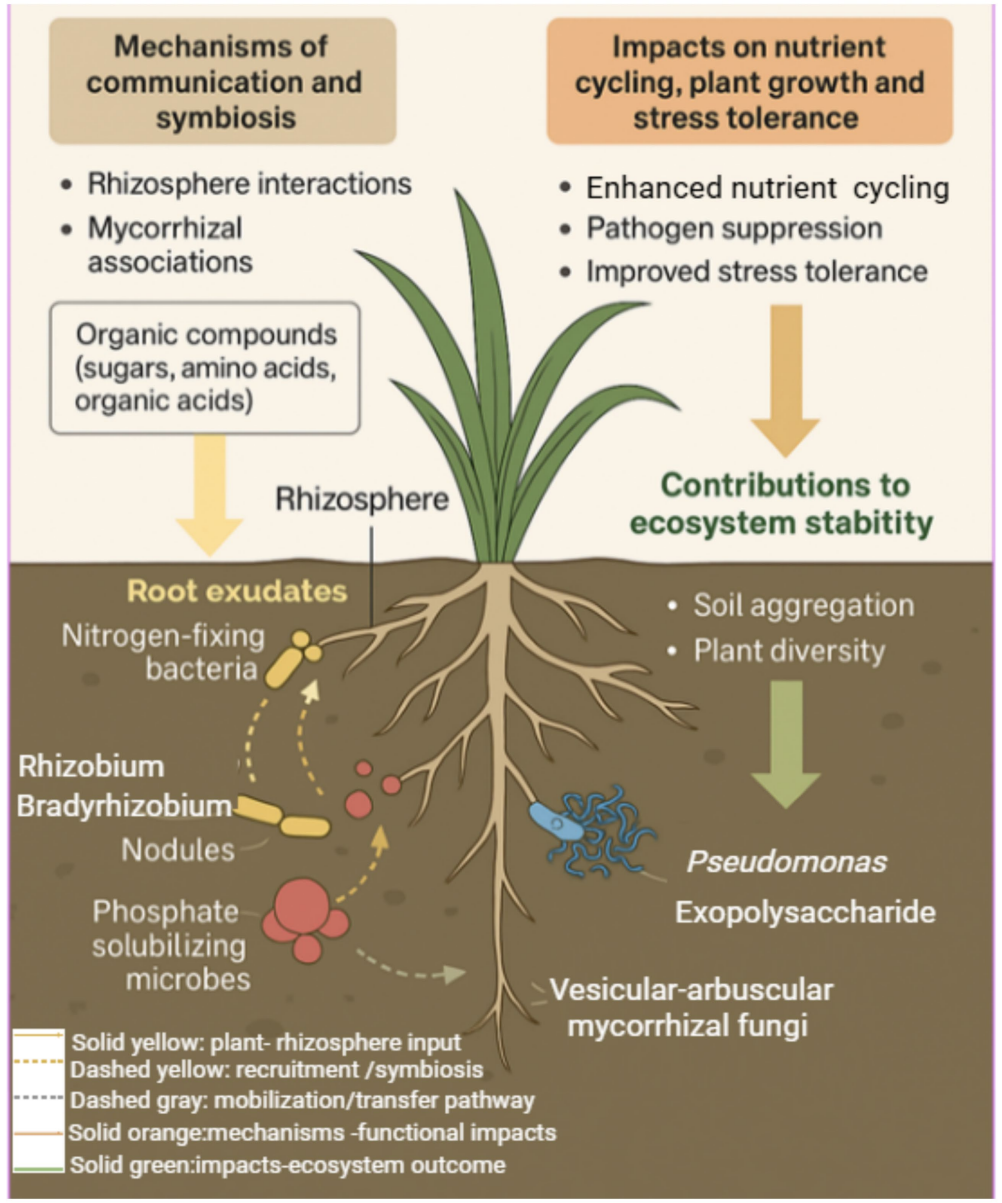
Conceptual model of soil–plant–microbe interactions in grassland ecosystems. The rhizosphere serves as a dynamic interface where root exudates, composed of sugars, amino acids, and organic acids, mediate communication and symbiosis between plants and soil microorganisms. Nitrogen-fixing bacteria (*Rhizobium* and *Bradyrhizobium*) form nodules, while phosphate-solubilizing microbes enhance phosphorus availability. VAMF and beneficial rhizobacteria such as *Pseudomonas* spp. contribute to nutrient acquisition and soil structural stability through hyphal extension and exopolysaccharide production. These interactions enhance nutrient cycling efficiency, suppress pathogens, and improve plant stress tolerance, ultimately promoting soil aggregation, plant diversity, and overall ecosystem stability in grasslands.

In grassland ecosystems, the interactions between soil, plants, and microbes are crucial for maintaining ecosystem functionality (Zhang R. et al., 2021; Zhang Z. et al., 2021). These interactions are often characterized by complex signaling and cooperative processes that enhance nutrient acquisition, stress resilience, and overall plant health. The main pathways include rhizosphere interactions, mycorrhizal associations, and chemical signaling between plants and soil microbiota (Figure 2).

Rhizosphere interactions, where plant roots and soil microbes directly interact, are foundational to plant growth and nutrient dynamics in grasslands (Garcia and Kao-Kniffin, 2018). Plants release a variety of organic compounds, such as sugars, amino acids, and organic acids, into the rhizosphere through their root exudates. These exudates serve as a carbon source for soil microbes, but they also act as signals to modulate microbial community structure and function (Carvalhais et al., 2011; Vives-Peris et al., 2020). Beneficial microorganisms, including nitrogen-fixing bacteria (*Rhizobium* and *Bradyrhizobium*) or *Pseudomonas* spp., are recruited through these exudates, subsequently promoting plant growth by producing phytohormones or enhancing nutrient availability (Jaiswal et al., 2021). This recruitment process forms a key element of the cooperative nutrient exchange between plants and microbes (Figure 2).

Mycorrhizal associations, particularly those involving VAMF, are another key form of symbiosis in grasslands (Neuenkamp et al., 2018). These fungi colonize plant roots and, in exchange for photosynthetically derived carbon, facilitate the uptake of essential nutrients, especially phosphorus, nitrogen, and micronutrients, which are often limiting in grassland soils (Neuenkamp et al., 2018). The extension of fungal hyphae into the rhizospheric soil greatly increases the effective root surface area, enabling plants to access otherwise unavailable nutrient pools (Figure 2). This mutualistic relationship enhances plant growth in nutrient-poor soils (Ellouze et al., 2014) and strengthens plant resistance to environmental stressors such as drought, salinity, and pathogen pressure (Nasim, 2010).

### 3.2 Impacts on nutrient cycling, plant growth, and stress tolerance

Plant–microbe interactions regulate nitrogen and phosphorus dynamics primarily through (i) root-exudate–mediated recruitment and activation of microbial guilds (Gupta et al., 2019), (ii) symbiotic nutrient exchange (especially mycorrhizal transfer), and (iii) microbially catalyzed transformations that control the chemical form and mobility of nutrients in the rhizosphere (Yadav et al., 2021).

For nitrogen, plants influence microbial activity by adjusting carbon allocation to roots and exudation profiles, which can stimulate diazotrophs and other N-transforming taxa and modulate nitrification/denitrification microzones via rhizosphere oxygen and moisture gradients (Huang et al., 2014). These “hotspots” determine when nitrogen is retained in plant-available forms versus lost through leaching or gaseous pathways (Kumar et al., 2025).

For phosphorus, plants and microbes interact through enzymatic and chemical mobilization (e.g., phosphatase activity and organic acid release) and through mycorrhizal hyphal networks that extend the nutrient foraging volume and directly transfer P to host plants. These mechanisms explain why similar restoration interventions can produce different outcomes depending on soil chemistry, resident microbiomes, and plant functional traits (Figure 2).

Beyond nutrient acquisition, plant–microbe interactions significantly improve plant stress tolerance. Mycorrhizal fungi enhance drought resilience by improving water uptake and maintaining plant physiological functions under water-limited conditions (Khan et al., 2021; Pattnaik et al., 2021). Plant growth–promoting rhizobacteria (PGPR), such as *Pseudomonas* spp., produce exopolysaccharides that improve soil aggregation, increase water retention, and stabilize soil structure under drought stress. They also secrete siderophores that enhance micronutrient availability and may induce systemic acquired resistance (SAR), increasing tolerance to both biotic and abiotic stress factors (Naseem et al., 2018; Kamle et al., 2020). The integration of these microbial strategies contributes to the overall resilience of grassland plant communities (Figure 2).

### 3.3 How these interactions contribute to ecosystem stability

Soil–plant–microbe interactions are central to the stability and sustainability of grassland ecosystems, influencing nutrient cycling, disease suppression, and soil fertility (Li et al., 2022). Microbial activity in the rhizosphere enhances nutrient availability while promoting soil aggregation and structural integrity (Chen et al., 2024). Extracellular polysaccharides produced by bacteria and fungi bind soil particles, increasing aggregate stability and improving porosity and water infiltration. These structural benefits enhance root penetration and water retention, particularly under drought stress (Ali et al., 2024). Such structural reinforcement is closely linked to the activity of root-associated microbial guilds (Figure 2).

In addition, rhizosphere microbial communities suppress soil-borne pathogens through competitive exclusion and the production of antimicrobial compounds. For example, *Bacillus* species inhibit pathogens such as *Fusarium* spp. and *Rhizoctonia* spp. through antibiotic production (Jangir et al., 2018). This natural disease suppression underpins plant health and helps maintain the diversity and functionality of grassland ecosystems (Teague and Kreuter, 2020).

Microbial interactions also promote plant biodiversity by facilitating nutrient acquisition, growth, and stress tolerance across diverse plant species (Baldi, 2021). This fosters more stable plant communities capable of withstanding disturbances such as drought and grazing, and of recovering rapidly after such events (Nadeem et al., 2014). By maintaining nutrient balance, preventing soil degradation, and supporting carbon sequestration, microbial diversity acts as a buffer against environmental fluctuations, thereby sustaining long-term grassland productivity and ecological resilience (Ploughe et al., 2019). In terms of ecosystem stability, microbial communities help maintain nutrient balances and prevent soil degradation (Figure 2). In grasslands, microbial diversity can act as a buffer, enabling ecosystems to adapt to changing environmental conditions, such as shifts in precipitation patterns or temperature regimes associated with climate change (Srivastava et al., 2023). These stable, functional microbial communities support the long-term productivity and health of grassland ecosystems, contributing to their ability to sequester carbon and support a wide range of plant and animal species (Bai and Cotrufo, 2022).

## 4 Microbiome responses to grassland degradation and disturbance

### 4.1 Effects of overgrazing, land-use change, climate change, and invasive species on microbial communities

**Figure 3:**
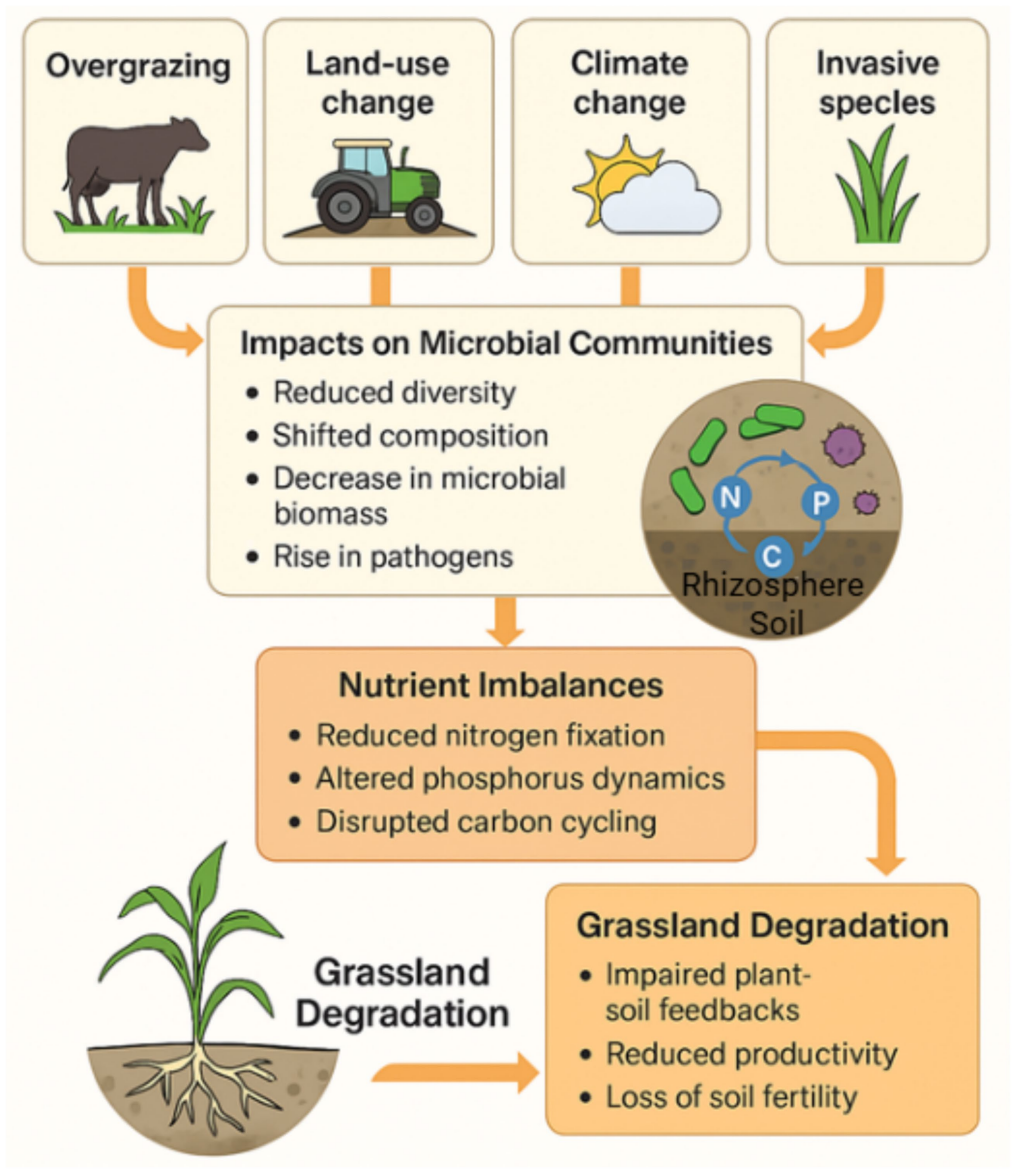
Microbiome responses to grassland degradation and disturbance. Overgrazing, land-use change, climate change, and invasive species alter soil microbial communities by reducing diversity, shifting taxonomic composition, decreasing microbial biomass, and increasing pathogen prevalence. These changes lead to nutrient imbalances, including reduced nitrogen fixation, altered phosphorus dynamics, and disrupted carbon cycling. The resulting nutrient limitations impair plant–soil feedbacks, reduce primary productivity, and cause loss of soil fertility, thereby accelerating grassland degradation.

Grasslands are highly sensitive ecosystems, and their microbial communities, particularly those in the rhizosphere and bulk soil, play a critical role in maintaining ecosystem function and resilience (Yuan et al., 2016). These microbiomes are integral to grassland functioning, with disturbances triggering structural and functional changes that propagate through the ecosystem (Figure 3). However, various anthropogenic and environmental disturbances including overgrazing, land-use change, climate change, and invasive species can negatively impact these microbiomes, leading to a cascade of degradation effects (De Vries et al., 2020).

Overgrazing is one of the most significant pressures on grasslands. Livestock grazing physically disturbs the soil and reduces vegetation cover, decreasing root biomass and altering the input of root exudates that sustain microbial life (Bell et al., 2011). Overgrazing is often associated with declines in beneficial microbial groups, such as nitrogen-fixing bacteria and mycorrhizal fungi. Additionally, it can lead to nutrient imbalances through soil compaction and erosion, which reduce nutrient availability and microbial habitat quality. For example, Song et al. (2022) reported that overgrazing decreased microbial diversity and promoted the growth of stress-tolerant and pathogenic taxa, inhibiting plant recovery. These structural shifts in microbial communities contribute directly to nutrient imbalances and subsequent soil degradation (Figure 3).

Land-use change, such as the conversion of native grasslands to croplands or urban areas, leads to significant shifts in microbial community structure (Wu et al., 2022). These changes typically result in reduced microbial diversity and favor opportunistic taxa adapted to disturbed and nutrient-enriched conditions. In the Loess Plateau, land conversion reduced microbial complexity and altered nutrient cycling by increasing the abundance of specific bacterial groups such as *Nitrobacter* and *Azotobacter* (Chen et al., 2022). This microbial restructuring disrupts nitrogen fixation and phosphorus cycling, reducing the soil’s capacity to support healthy grassland vegetation (Figure 3).

Climate change introduces additional stressors to microbial communities in grasslands, including altered temperature and precipitation patterns. These changes can shift microbial composition and function, particularly through soil moisture and temperature regime alterations (Zhang et al., 2016). Elevated temperatures may promote thermophilic and drought-tolerant microbes while suppressing taxa adapted to cooler, more stable environments. Likewise, prolonged droughts can reduce microbial biomass and decrease the abundance of decomposers, limiting organic matter turnover (Bastida et al., 2017). These climate-driven changes can be further compounded by invasive species, which disrupt native plant–microbe associations and introduce novel pathogens (Figure 3). For example, the invasive grass *Bromus tectorum* alters soil microbial communities by increasing fungi that promote its own growth while reducing the diversity of microbes supporting native species in Western U. S. grasslands (Finch et al., 2021).

### 4.2 Microbial indicators of grassland degradation

Soil microbial diversity serves as a critical metric for evaluating grassland ecosystem integrity, functioning, and resilience (Shu et al., 2023). The loss of microbial diversity under disturbance conditions has been extensively documented and is now widely accepted as a hallmark of ecosystem degradation (Sharma et al., 2010). In grasslands, microbial communities contribute to nutrient acquisition, organic matter turnover, and pathogen resistance. High diversity ensures functional redundancy, enabling ecosystems to buffer environmental fluctuations. Conversely, when disturbances such as overgrazing, drought, or land-use change reduce microbial diversity, the system becomes more vulnerable to ecological collapse (Pedrinho et al., 2024). These declines are often accompanied by reductions in network complexity and the fragmentation of microbial interactions (Ploughe et al., 2019). The resulting simplification reduces the functional potential of the microbiome, weakening plant nutrient uptake and increasing susceptibility to stressors (Bardgett et al., 2021). Such patterns correspond to broader degradation pathways involving nutrient imbalance and soil structural decline (Figure 3).

Functional indicators also highlight the extent of microbial decline. Reduced enzymatic activity—including phosphatases, dehydrogenases, and ureases—has been reported in degraded grasslands (Du et al., 2024), while functional gene profiling often reveals declines in nitrogen cycle–related genes (e.g., *nifH*, *amoA*, and *nirK*) in disturbed environments. These reductions point to impaired nutrient transformations and diminished resilience. Beta diversity analyses reveal a trend toward microbial homogenization along degradation gradients (Solomon, 2015; Zhang et al., 2017), indicating fewer ecological niches and less spatial heterogeneity in degraded systems. Elevated bacterial-to-fungal ratios, alongside declines in fungal-mediated nutrient cycling, also serve as quantitative degradation markers (Liu-Xu et al., 2024). Enzyme activity loss and pathogen dominance, such as increases in *Fusarium* spp. or *Rhizoctonia* spp., signal shifts toward less beneficial community compositions (Boeddinghaus et al., 2019), further reinforcing the degradation trajectory (Figure 3).

### 4.3 Feedback loops between microbiome shifts and grassland decline

Microbial communities drive key nutrient cycles, nitrogen, phosphorus, and carbon, and their disruption can trigger cascading imbalances that accelerate grassland degradation (Sveen et al., 2024). In nitrogen cycling, the decline of nitrogen-fixing bacteria reduces atmospheric nitrogen inputs, while disruptions in nitrifying and denitrifying communities cause imbalances such as nitrate accumulation or nitrogen gas loss. The loss of *Nitrosospira* and *Nitrobacter* reduces nitrification efficiency (Yuan et al., 2016), while incomplete nitrification can elevate soil ammonia to toxic levels, impairing root development (Figure 3).

Phosphorus availability is similarly affected. In healthy grasslands, microbial phosphatases release inorganic phosphorus from organic matter, but in degraded soils, reduced microbial biomass and enzyme activity hinder this process (Katsalirou et al., 2016). The decline of phosphate-solubilizing microbes, including *Penicillium*, *Aspergillus*, and *Pseudomonas*, further restricts phosphorus bioavailability (Lin et al., 2022). Carbon cycling is also disrupted: reduced decomposition limits soil organic matter turnover, while erosion and leaching remove labile carbon fractions (de Nijs and Cammeraat, 2020). These interconnected nutrient disruptions feed back into reduced vegetation cover, lowering root exudation and further depleting microbial communities (Figure 3).

Invasive species add another feedback dimension by promoting microbial assemblages that favor their own growth while suppressing beneficial symbionts of native flora (Rout and Callaway, 2012). This shift reduces soil resilience and impedes native plant recovery, locking degraded systems into invasive-dominated states (Batten et al., 2008; Chen et al., 2020).

### 4.4 Conceptual integration: microbial pathways to grassland degradation

The conceptual model in Figure 3 synthesizes the disturbance–microbiome–ecosystem feedback framework for grasslands. Overgrazing, land-use change, climate variability, and invasive species disrupt microbial diversity, composition, and function, initiating nutrient imbalances, functional gene losses, and pathogen proliferation. Disturbance-driven microbiome shifts can alter the direction and strength of plant–soil feedbacks, often enhancing negative PSFs via pathogen accumulation and loss of mutualists, while reducing positive PSFs that support nutrient acquisition and stress tolerance; thus, the net effect on grassland health depends on whether PSFs become predominantly harmful or beneficial (Kumar et al., 2025).

Crucially, these processes operate in reinforcing loops: loss of beneficial microbes reduces nutrient uptake and plant vigor, leading to less root-derived carbon entering the soil, which in turn exacerbates microbial decline. Amplifying factors such as invasive plant dominance and climate extremes intensify these feedbacks, pushing the system toward long-term soil infertility and productivity loss (Guo et al., 2019). Recognizing these microbial mechanisms enables targeted restoration strategies that focus on rebuilding microbial diversity, reintroducing beneficial taxa, and restoring functional nutrient cycles as a pathway to reversing grassland degradation (Figure 3).

## 5 Harnessing microbiomes for grassland restoration

### 5.1 Microbial inoculants: biofertilizers and mycorrhizal fungi for soil and plant health

**Figure 4:**
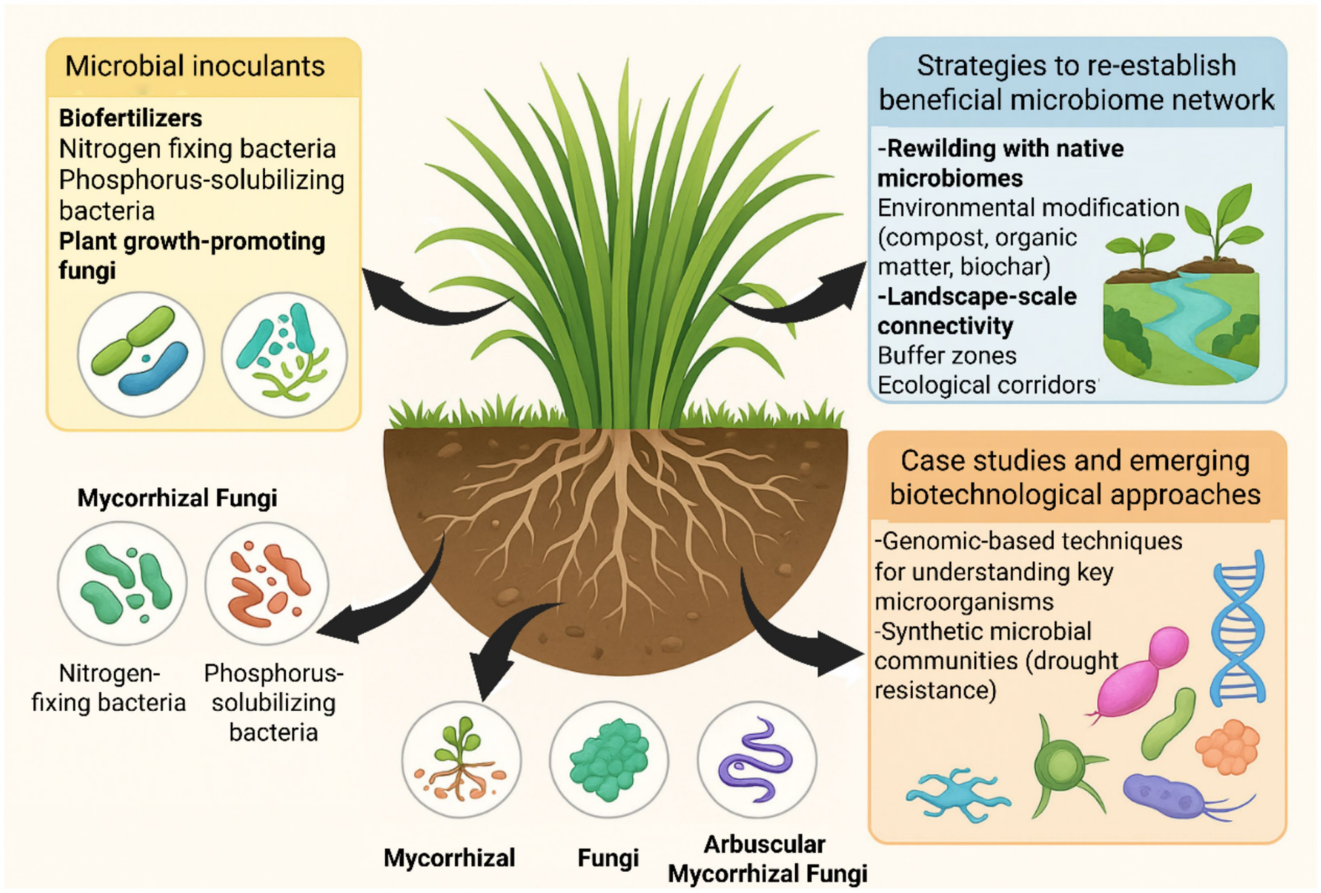
Harnessing microbiomes for grassland restoration. Overview of microbiome-based strategies for restoring degraded grasslands. Biofertilizers and mycorrhizal fungi enhance nutrient cycling, plant growth, and stress tolerance. Rewilding with native microbiomes, organic amendments, and improved landscape connectivity support the re-establishment of functional microbial networks. Emerging approaches, including genomic tools and synthetic microbial communities, further optimize drought resistance, nutrient cycling, and soil structure, promoting long-term ecosystem resilience.

Harnessing beneficial soil microbiomes through microbial inoculants represents a promising, ecologically sound strategy for restoring degraded grasslands (Yuan et al., 2025). Inoculants such as biofertilizers and mycorrhizal fungi enhance plant health, nutrient cycling, and soil structure key components of ecosystem recovery (Jagadesh et al., 2024). These microbial inputs are designed to supplement or restore the native microbial community, thereby catalyzing plant-microbe interactions essential for ecosystem functionality (O’Callaghan et al., 2022). The principal groups of microbial inoculants and their restoration roles are summarized in Figure 4.

Biofertilizers, commonly composed of nitrogen-fixing bacteria (e.g., *Rhizobium* spp.), phosphorus-solubilizing bacteria (e.g., *Bacillus* spp.), and plant growth-promoting rhizobacteria (PGPR), have gained attention for their ability to enhance nutrient availability and stimulate plant growth (Aloo et al., 2022). These organisms contribute to essential processes such as nitrogen fixation and phosphorus solubilization, addressing nutrient limitations that are often a hallmark of degraded soils. For example, Yadav et al. (2021) demonstrated that inoculation of native grasses with nitrogen-fixing bacteria significantly improved plant biomass and soil nitrogen content in overgrazed grasslands, thereby promoting overall ecosystem productivity. In addition to nutrient enhancement, biofertilizers have been shown to improve plant stress tolerance under abiotic stressors such as drought, salinity, and low fertility (Bhardwaj et al., 2014).

Mycorrhizal fungi are another class of microbial inoculants with substantial potential in grassland restoration. These fungi form symbiotic associations with plant roots, enhancing the uptake of poorly mobile nutrients (particularly phosphorus) while simultaneously increasing plant resistance to environmental stresses (Koziol and Bever, 2017). Inoculation with native mycorrhizal fungi has been shown to improve soil microbial diversity, enhance plant growth, and increase plant species richness in degraded landscapes (Zhang R. et al., 2021; Zhang Z. et al., 2021). Their extensive hyphal networks also contribute to improved soil aggregation, water retention, and nutrient flow, reinforcing soil structural stability and vegetation cover (Figure 4). By facilitating nutrient exchange and improving plant health, these fungi play a central role in restoring ecosystem functions in degraded grasslands (Ding et al., 2024).

Together, microbial inoculants such as biofertilizers and mycorrhizal fungi represent cost-effective and environmentally sustainable tools to restore the ecological functions of grasslands. Their application not only improves soil quality and plant productivity but also enhances long-term ecosystem services, including carbon sequestration, erosion control, and biodiversity support (Jagadesh et al., 2024).

Overall, microbial inoculation and organic amendments offer clear advantages for grassland restoration, as they accelerate nutrient acquisition, improve soil structure, and enhance plant stress tolerance (Han et al., 2025). However, outcomes are frequently context-dependent, shaped by soil properties, climate, resident microbiomes, and plant community composition (Liu et al., 2024). Key gaps include inconsistent persistence of introduced strains, limited understanding of non-target effects on native microbial networks, and the lack of standardized field protocols linking inoculation/amendment choices to measurable functional gains. Future work should prioritize locally adapted consortia, mechanistic validation (process rates and functional genes), and long-term monitoring to assess durability and ecological safety.

### 5.2 Strategies to re-establish beneficial microbiome networks

Restoring degraded grasslands requires more than improving soil fertility; it demands the re-establishment of functional microbiome networks that underpin ecosystem stability (Yang et al., 2025). These networks comprise diverse microbial taxa, including bacteria, archaea, and fungi that synergistically mediate nutrient cycling, plant growth, and stress resilience (Peddle et al., 2025). Core strategies for rebuilding such networks are depicted in Figure 4.

Rewilding degraded soils with native microbiomes is a promising approach. This involves introducing microbial communities sourced from healthy, undisturbed grasslands into degraded sites (Bijl et al., 2011). Such inoculation strategies can restore microbial diversity, re-establish plant-microbe symbioses, and accelerate ecosystem recovery (Wang et al., 2024). For example, Liao et al. (2023) reported that soil inoculation with microbial consortia derived from reference grasslands significantly improved plant community composition and functional traits in disturbed ecosystems. The use of native microbial communities also ensures better ecological compatibility and minimizes the risk of disrupting local biotic interactions (Yadav et al., 2021).

Enhancing plant–microbe interactions through environmental modification is another effective strategy. Soil amendments such as compost, organic matter, and biochar can improve soil physicochemical properties, stimulate microbial activity, and create favorable conditions for beneficial symbioses (Anas et al., 2025). Organic amendments serve as substrates for microbial colonization and enhance soil structure, aeration, and nutrient-holding capacity. Such environmental modifications provide the structural and chemical conditions needed for microbiome re-establishment (Figure 4). Wen et al. (2024) demonstrated that biochar addition to degraded grassland soils increased microbial biomass, promoted mycorrhizal colonization, and improved native plant growth.

Landscape-scale connectivity also plays a pivotal role in microbial restoration. Enhancing connectivity between degraded and intact ecosystems facilitates microbial dispersal, increases gene flow, and promotes the recolonization of beneficial taxa (Mutillod et al., 2024). Practices such as establishing buffer zones, ecological corridors, and reducing landscape fragmentation enable microbial communities from reference systems to naturally migrate and establish in restored sites (McKinley, 2019). These measures enhance microbial diversity and functional redundancy, key determinants of ecosystem stability and resilience.

### 5.3 Case studies and emerging biotechnological approaches

Numerous case studies and emerging biotechnological approaches have demonstrated the potential of microbiomes in grassland restoration. These studies highlight the importance of integrating microbiome-based strategies into ecological restoration practices, with promising results in both small-scale and large-scale restoration projects (Robinson et al., 2023).

One example is the restoration of degraded grasslands in China’s Loess Plateau, where microbial communities from healthy grasslands were introduced into degraded sites (Guo et al., 2019). Research conducted by Zhang et al. (2024) showed that such inoculation significantly improved plant diversity, biomass, and soil fertility, with microbial communities shifting toward nutrient-cycling assemblages that enhance resilience. These outcomes align with the rewilding and inoculation strategies outlined in Figure 4.

Emerging biotechnological approaches are expanding the scope of microbiome-based restoration. Metagenomic sequencing and synthetic microbiology are being used to identify key microorganisms and microbial consortia that can be engineered or introduced into grassland ecosystems to restore microbial balance (Kennedy et al., 2007). For instance, a recent study by Hao et al. (2023) explored synthetic microbial communities designed to promote drought resistance and nutrient cycling in degraded grasslands. By combining bacteria, fungi, and archaea with complementary functions, they enhanced nitrogen fixation, plant drought tolerance, and soil structure. This approach demonstrates how tailored microbial communities can be optimized for site-specific restoration needs (Fry et al., 2018).

Advances in genomic-based techniques have also deepened our understanding of the functional roles of specific microbial taxa. Sequencing the genomes of key nutrient-cycling and plant-beneficial microorganisms has revealed novel traits for potential use in restoration (Wellington et al., 2003). For example, the identification of new phosphate-solubilizing bacterial species has informed the development of targeted inoculants for improving phosphorus availability in nutrient-poor soils (Ughamba et al., 2025). These advances, integrated with ecological strategies, provide a robust framework for microbiome-based grassland restoration (Thakur et al., 2023).

## 6 Future directions and conclusions

### 6.1 Research gaps in soil–plant-microbe interactions in grasslands

Despite a growing body of research demonstrating the ecological importance of soil–plant–microbe interactions in grassland ecosystems, several critical knowledge gaps persist that constrain the development of microbiome-informed restoration strategies. One of the most pressing gaps is the insufficient understanding of spatiotemporal variability in microbial communities across grassland types and climatic zones (Nizamani et al., 2024). Microbial assemblages are known to shift with factors such as soil type, vegetation composition, topography, and seasonal dynamics; however, systematic, long-term studies capturing these variations are scarce. Temporal monitoring across restoration chronosequences and disturbed gradients is necessary to elucidate the successional trajectories of microbial communities and identify the biotic and abiotic drivers of microbial assembly and function (Guo et al., 2022).

Equally underexplored is the functional role of specific microbial taxa and guilds in supporting ecosystem processes under restoration. While the contributions of well-known groups such as nitrogen-fixing bacteria and mycorrhizal fungi are documented, many other functionally important taxa such as phosphate-solubilizing bacteria, ammonia oxidizers, sulfate reducers, and microbial predators remain poorly characterized in the context of grassland health (Chen et al., 2024). Leveraging multi-omics technologies, including metagenomics, metatranscriptomics, and stable isotope probing, can advance understanding of functional microbial diversity and its contributions to nutrient cycling, disease suppression, and abiotic stress mitigation (Satya et al., 2024). In addition, the responses of microbial networks to emerging environmental pressures such as climate change, invasive plant species, and altered land-use regimes remain insufficiently understood. These stressors can destabilize microbial networks and disrupt keystone symbioses critical for plant fitness and ecosystem resilience. Disentangling the mechanisms of microbial adaptation and community resilience under environmental disturbances will be essential for predicting and enhancing the success of grassland restoration efforts under future climate scenarios.

### 6.2 Advancing microbiome engineering in restoration ecology

Microbiome engineering has emerged as a promising frontier in restoration ecology, offering targeted strategies to enhance the ecological functionality of degraded grasslands. This approach involves manipulating microbial communities through the application of beneficial taxa, synthetic microbial consortia, or habitat modifications to optimize microbial-mediated ecosystem functions such as nutrient availability, soil structure formation, and pathogen resistance (Hao et al., 2023).

Recent advances in synthetic biology and microbial ecology have enabled the rational design of microbial consortia tailored for specific restoration objectives, including the promotion of drought-tolerant plant species or acceleration of nutrient cycling (Peddle et al., 2025). Additionally, functional gene screening through metagenomics provides insights into microbial traits linked to key ecosystem functions, offering molecular targets for microbial selection or genetic enhancement.

Cutting-edge technologies such as CRISPR-Cas-mediated genome editing hold the potential to engineer microbial strains with enhanced metabolic capabilities, including the degradation of pollutants, synthesis of plant growth-promoting compounds, or resilience to abiotic stresses (Sahoo et al., 2022). However, such interventions necessitate rigorous risk assessments. The ecological risks associated with introducing engineered or non-native microbial strains, such as competitive exclusion of native microbiota, horizontal gene transfer, or unintended trophic interactions, require careful evaluation through controlled field trials and long-term monitoring frameworks (Chemla et al., 2024).

To realize the full potential of microbiome engineering, interdisciplinary collaboration is imperative, bridging microbiology, restoration ecology, molecular biology, and regulatory science to develop ecologically responsible and scalable solutions (Robinson et al., 2023).

### 6.3 Integrating microbiome knowledge into grassland management frameworks

Integrating microbiome science into practical grassland management represents a transformative step toward sustainable restoration. Achieving this requires a shift from traditional, plant-centric paradigms to a more holistic approach that recognizes microbial communities as central agents of ecological recovery and system stability.

One pathway for integration is through adaptive management frameworks that incorporate microbiome-informed indicators and feedback mechanisms into restoration planning and evaluation. For instance, the characterization of microbial community composition, diversity, and functional potential can serve as diagnostic tools to assess ecosystem recovery or degradation trends. High-throughput sequencing and bioinformatics pipelines now allow for the rapid profiling of microbial biomarkers associated with soil health, nutrient cycling, or restoration success.

Practical applications of microbiome-informed management may include customized microbial inoculants, adjusted seeding protocols to support symbiotic microbial recruitment, and the use of organic soil amendments that foster beneficial microbial growth. Moreover, land-use decisions such as grazing regimes, fire management, or irrigation should be guided by an understanding of their downstream effects on microbial communities.

Importantly, integrating microbiome science into management frameworks requires stakeholder engagement and capacity-building, particularly among land managers, restoration practitioners, and policy makers. Developing accessible tools, decision-support systems, and microbiome-based guidelines will help translate scientific findings into actionable practices.

### 6.4 Conclusion

Grassland microbiomes (bacteria, fungi, archaea, and viruses) are central to ecosystem functioning by regulating nutrient cycling, soil aggregation, organic matter turnover, and plant health. Evidence from degradation gradients and restoration efforts consistently indicates that disturbance reduces microbial diversity and network complexity, disrupts functional guilds (e.g., nitrogen fixers and mycorrhizae), and can increase pathogen dominance, ultimately constraining vegetation recovery and ecosystem stability.

Despite rapid progress in microbiome characterization, major gaps remain in linking community shifts to causal mechanisms and measurable process rates, in resolving spatiotemporal variability across grassland types, and in incorporating underrepresented yet influential groups (e.g., viruses and microbial predators) into restoration theory and monitoring. In addition, the effectiveness and persistence of microbial inoculants and organic amendments remain context-dependent, and standardized field protocols and indicators for “restoration-ready microbiomes” are still lacking.

Future research should integrate multi-omics with process-based measurements (e.g., enzyme activities, nutrient transformation rates, stable-isotope tracing) across long-term chronosequences, while explicitly considering viral life-history strategies and their ecosystem implications. Translational priorities include developing microbiome-informed indicators for adaptive management, testing locally adapted microbial consortia with long-term monitoring, and strengthening ecological risk assessment and governance frameworks for engineered or introduced microorganisms.
