# Correction: From microbiome collapse to recovery: a roadmap for microbiome-informed grassland restoration under global change

**DOI:** 10.3389/fmicb.2026.1819081

**Published:** 2026-03-10

**Authors:** Xu Qiao, Xu Yan, Cui Dong, Lin Tao, Aishajiang Aili, Abdul Waheed

**Affiliations:** 1 College of Resources and Environment, Yili Normal University, Yining, Xinjiang, China; 2 Institute of Resources and Ecology, Yili Normal University, Yining, Xinjiang, China; 3 Ecological Water Conservancy Research Center of Xinjiang Uygur Autonomous Region, Ürümqi, China; 4 Xinjiang Comprehensive Land Consolidation and Rehabilitation Center, Ürümqi, China; 5 Technology Innovation Center for Ecological Monitoring and Restoration of Desert-Oasis, MNR, Ürümqi, China; 6 State Key Laboratory of Desert and Oasis Ecology, Xinjiang Institute of Ecology and Geography, Chinese Academy of Sciences, Ürümqi, China

**Keywords:** ecosystem resilience, grassland ecosystems, grassland restoration, microbial diversity, microbiome engineering, soil–plant interactions

In the published article, there was an error in the **Generative AI statement**. The published statement indicated that Generative AI was not used, whereas Generative AI was used to assist in preparing the illustrative diagrams in Figures 1–4.

A correction has been made to the Generative AI statement, Paragraph 1. This sentence previously stated:

“The author(s) declared that Generative AI was not used in the creation of this manuscript.”

**The corrected sentence appears below:**

“The author(s) declared that Generative AI was used in the creation of this manuscript. The author(s) declared that Generative AI was used in the preparation of the illustrative diagrams in Figures 1–4 to assist with visual design and layout. All AI-assisted outputs were reviewed and edited by the authors. Generative AI was not used for data analysis, data interpretation, or the scientific conclusions of the manuscript.”

The original article has been updated.

